# Topology-based segmentation of 3D confocal images of emerging hematopoietic stem cells in the zebrafish embryo

**DOI:** 10.1017/S2633903X24000102

**Published:** 2024-11-11

**Authors:** G. Nardi, L. Torcq, A. A. Schmidt, J.-C. Olivo-Marin

**Affiliations:** 1 Biological Image Analysis Unit, Institut Pasteur, Université Paris Cité, Paris, France; 2 CNRS UMR3691, Paris, France; 3 Department of Developmental and Stem Cell Biology, Institut Pasteur, Université Paris Cité, Paris, France; 4 CNRS UMR3738, Paris, France; 5 Collège doctoral, Sorbonne Université, Paris, France

**Keywords:** 3D segmentation, morphogenesis, persistent homology, cell evolution, meshing

## Abstract

We develop a novel method for image segmentation of 3D confocal microscopy images of emerging hematopoietic stem cells. The method is based on the theory of persistent homology and uses an optimal threshold to select the most persistent cycles in the persistence diagram. This enables the segmentation of the image’s most contrasted and representative shapes. Coupling this segmentation method with a meshing algorithm, we define a pipeline for 3D reconstruction of confocal volumes. Compared to related methods, this approach improves shape segmentation, is more ergonomic to automatize, and has fewer parameters. We apply it to the segmentation of membranes, at subcellular resolution, of cells involved in the endothelial-to-hematopoietic transition (EHT) in the zebrafish embryos.

## Impact Statement

This article develops a novel method to perform 3D segmentation of confocal volumes, enabling a topologically consistent mesh representation. In particular, the proposed pipeline allows accurate reconstruction of relevant subcellular structures, a key advantage of 3D modeling without ground truth. This article results from a collaboration with developmental biologists studying the morphogenesis of hematopoietic stem cells emerging from the aortic wall in the zebrafish embryo. In this context, robust segmentation algorithms are needed for 3D visualization of cellular evolution for ultimate biophysical modeling.

## 1. Introduction

Object segmentation is a central field of image processing, and many methods have been proposed in the last decades. The main approaches consist of intensity thresholding,^1^ pixel clustering,^2^ or variational models to define optimal contours.^3^ ^,^^4^ However, to cope with the specificities of acquisition techniques, *ad hoc* routines are often designed to handle both the signal characteristics and the geometric properties of the shapes to be segmented.

This can be achieved by image pre-processing to enhance slices’ segmentation and 3D consolidation^5^ ^,^^6^ or by imposing an *a priori* knowledge of the 3D geometry.^7^ ^,^^8^ In Ref. 9, 3D Otsu thresholding and morphological operations allow the detection of membrane motifs such as filopodia and blebs. In Ref. 10, cell division is investigated in 3D by a previously developed algorithm^11^ based on manual initialization and deformable mesh optimization. Recently, the advent of deep learning has allowed the development of high-performance methods for several applications.^12^ ^–^^14^ However, these methods require large datasets and ground truths, which are rarely available for newly studied problems.

In this context, the theory of persistent homology^15^ provides a set of powerful techniques for shape detection within images. Objects can be tracked within the intensity graph based on the topological analysis of intensity-level sets, and topological features are detected with an associated lifetime (persistence). This information is contained in the persistence diagram, which defines a topological inventory of the image objects, allowing discrimination based on related persistence. We notice that the topology characterization of spaces relies on topological invariants, which, in dimension two, correspond to the number of connected components and holes. This makes this approach particularly suitable for the detection of disconnections and voids.

Persistent homology theory has many advantages compared to the standard segmentation methods. First, the topological analysis performed on the intensity graph allows the detection of all structures with associated persistence and circumvents the problem of defining optimal intensity thresholds. Additionally, the persistence diagram summarizes the topological structure of images, allows the selection of relevant structures via persistence thresholding, and defines a topological signature of the image. Finally, some stability results hold for persistence diagrams,^16^ ^,^^17^ proving that similar functions (in the 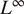 sense) have similar diagrams (in terms of bottleneck or Wasserstein metric).

Persistent homology is increasingly used in image processing to perform object detection^18^ and segmentation.^19^ ^–^^21^ In neural network methods, topological regularization terms have also been introduced to preserve topological features during learning.^22^ ^,^^23^ Finally, as persistence diagrams define topological image signatures, their comparison provides new machine learning approaches for tissue characterization.^24^ ^,^^25^

This work proposes a framework for a novel topology-based method for 3D segmentation of volume images of emerging hematopoietic stem cells. The approach is based on slice segmentation and mesh reconstruction. An optimal persistence threshold is applied to the slice’s persistence diagram to detect the main topological features. This identifies biological structures, such as folds and cavities, which allow realistic 3D reconstruction. Specific rules are defined to refine nested detections, helping to avoid splitting and branching issues during mesh reconstruction. Finally, an adapted tiling method is defined to reconstruct the 3D segmentation. Several results show that the proposed method outperforms standard segmentation methods.

The proposed method is applied to confocal volumes, showing the endothelial-to-hematopoietic transition (EHT) of the aorta’s endothelial cells in zebrafish. This type of cell emergence process has been studied qualitatively in recent works,^26^ ^–^^28^ but the lack of a 3D model prevents any quantitative approach. Confocal imaging enables the exploration of the longitudinal sections of subcellular structures, pointing out complexity in geometrical evolution, with increasing bending of luminal and basal membranes, the complex dynamics of the former, as well as cytoplasmic voids. These morphological motifs appear as dark cavities within longitudinal sections and are the only reliable information about the corresponding 3D shape. However, the lack of ground truth prevents the definition of topological or geometrical segmentation or meshing rules. Then, precise segmentation of the membrane profile and its internal holes is primordial for consistent 3D visualization. This enables the identification of different internal structures (for instance, cytoplasmic cavities and nucleus) and characteristic patterns in the membrane profile (for instance, bending, folding, and luminal invagination). Observing the different structures over time facilitates the visualization of the cell evolution and understanding the biophysical constraints governing it. In particular, the detection of internal cavities enables a consistent 3D reconstruction of the cell architecture, allowing distinguishing luminal invagination and internal voids.

This article is organized as follows. Section 2 introduces the main properties of persistent homology theory and shows how it can be applied to images. Section 3 defines the 3D segmentation method based on persistence thresholding and the mesh reconstruction algorithm. Finally, in Section 4, we present some applications to real data. First, we briefly introduce the EHT and present its geometrical characteristics on real data. In the second part, we show the results of our method on related images. Comparative tests show that our approach outperforms standard segmentation methods, and some examples of 3D reconstruction are shown.

## 2. Introduction to persistent homology for images

This section briefly exposes the framework of simplicial homology and persistent homology. We limit this exposition to definitions and results related to applications in image processing. For a complete and more formal presentation of these theories, we refer to Ref. 15.

### 2.1. Homology groups

The notion of homology group enables a formal algebraic definition of a space’s topological invariant (preserved by homeomorphism). In dimension two, these invariants are the number of its connected components and holes, corresponding to Betti’s number of order zero and one, respectively.

Homology theory can be established in the setting of a cubical complex, which is suitable for application to image processing. We remember that a cubical complex 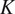 is a set of topologically simple pieces, called simplices, of different dimensional order and whose intersections are lower-dimension pieces still belonging to 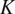 . Geometrically, we can consider points as 0-dimensional pieces, lines connecting two points as 1-dimensional simplices, and the squares between connected points as 2-dimensional simplices of the space.

Interestingly, topological invariants are related to the set of curves of a given order 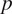 , called 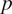 -chain, defined on the simplicial complex. For a given 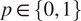 _,_ we can define two types of 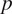 -curves: 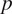 -cycles corresponding to curves with empty boundaries and 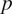 -boundaries representing the boundaries of a 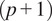 subcomplex. Roughly speaking, the interior of 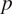 cycles can contain 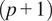 -dimensional voids.

The homology group 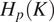 is then defined as the quotient group of the 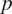 -cycles group by the *p*-boundary one. Homology classes thus define cycles that are not boundaries. In the case of 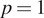 , which concerns the following, a representative of a homology class is a curve going around a hole in the complex.

### 2.2. Persistent homology

Persistent homology studies how homology groups and respective homology classes vary along the level set of a function defined on a simplicial complex 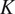 . As detailed below, this allows pointing out level sets’ main topological structures and helps distinguish between geometrical features and noise.

Let 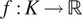 be a monotonic function defined on a simplicial complex 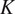 taking a finite set of values 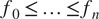 . In this context, monotonicity means that, for each element of 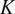 , the function takes equal or lower values on its boundary. Then, we can consider the family of sets 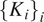 _,_ where 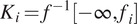 is a subcomplex of 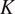 . Such a family is called filtration of 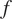 and verifies 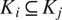 if 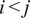 .

As the level sets are nested, there exists an induced homeomorphism on the respective homology groups 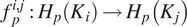 for 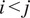 and 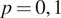 . This allows the tracking of homology classes from one set to another, verifying if a class at the level 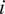 is still present at the level 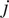 . Geometrically speaking, for 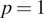 , this is equivalent to verifying if the hole represented by this class at the level 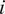 is still present in the level set 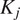 . Then, homology classes can persist, appear, or disappear from one level set to another depending on the relative hole’s persistence, opening, or merging. The case 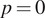 provides a similar analysis of connected component evolution through filtration.

In particular, we say that a class 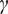 is born at 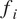 if 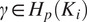 and 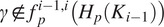 . Moreover, if a class 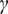 is born at 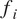 , we say that it dies at 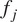 if it disappears entering in 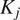 , which means that 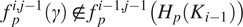 but 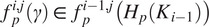 .

The difference between the function values of birth and death is called the persistence of the class:

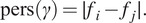

In this way, it is possible to follow every homology class (every hole for 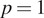 ) and determine which classes are the more persistent. The idea behind this approach is that persistent classes represent meaningful features of the complex, while the less persistent ones represent the noise.^29^

**Figure 1.**
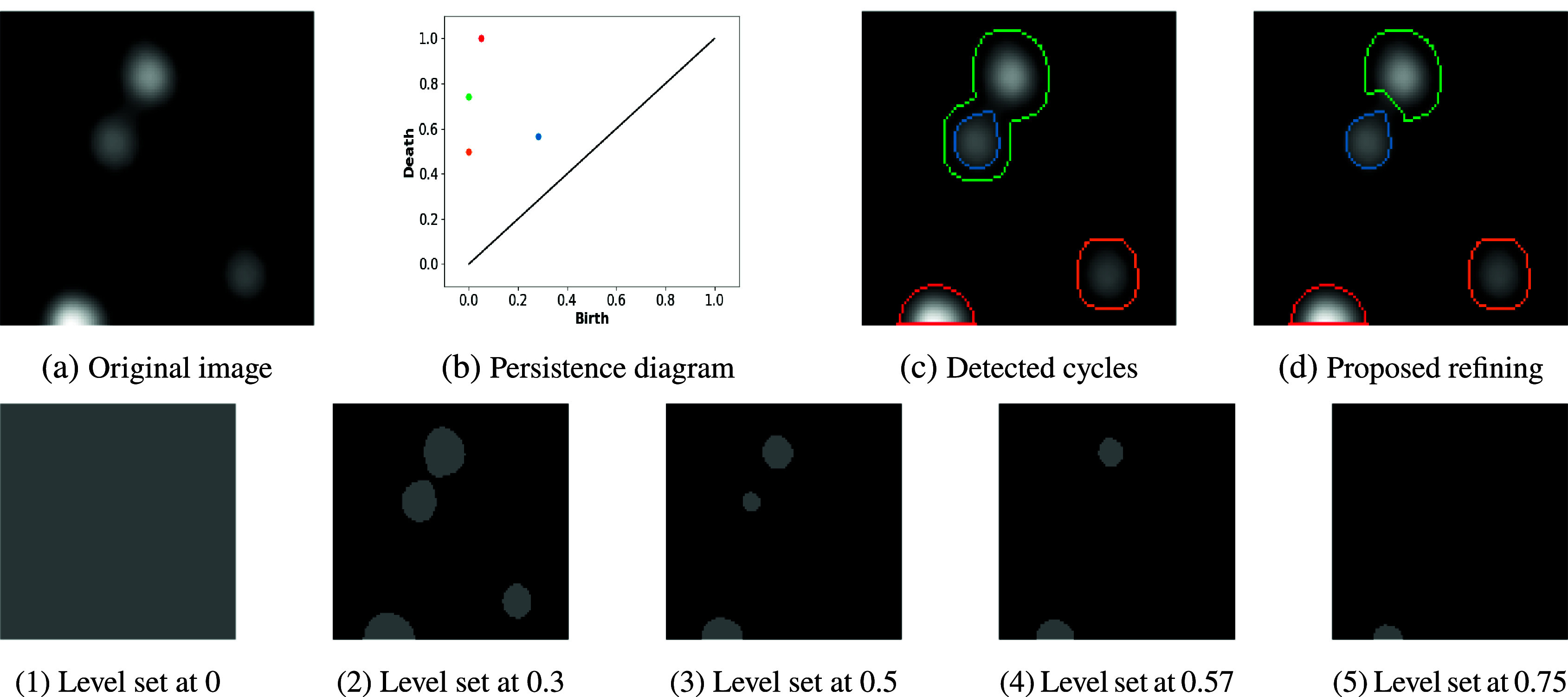
Filtration of lower-level sets and segmentation based on persistent diagram. Top panel: different steps of cycle segmentation based on the persistent diagram: (b) and (c) show the correspondence between points of the persistent diagram and related representatives, (d) shows the result of the refinement method for nested cycles proposed in this work. Bottom panel: image-level sets corresponding to birth and death intensity values of cycles belonging to the persistent diagram.

It is usual to display the couples of birth and death of each class on a persistence diagram. This facilitates the analysis of the topological structure of the graph of 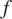 by associating to each class a point whose distance from the diagonal line coincides with its persistence. An example is given in Figure 1.

**Figure 2.**
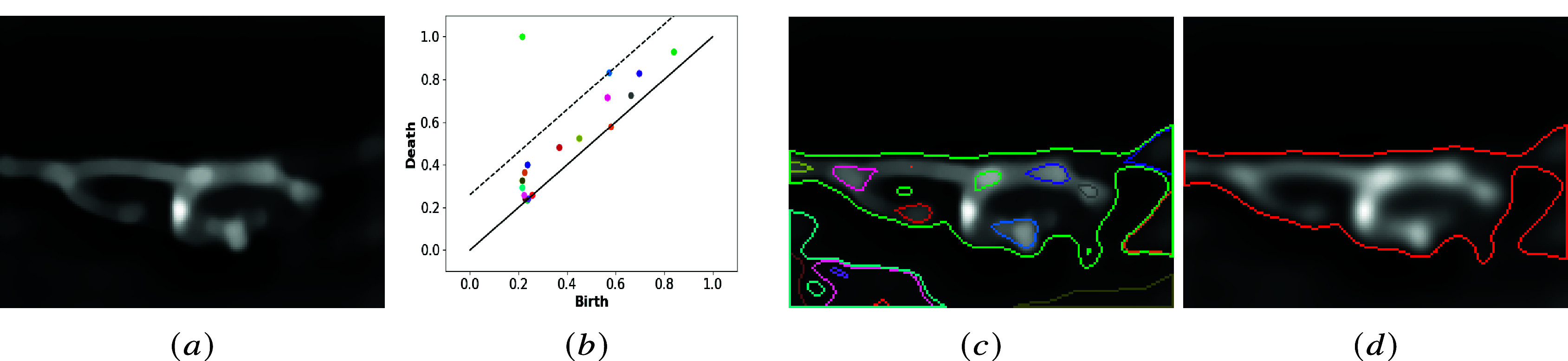
Segmentation of cell membranes: (a) the deconvolved original image; (b) persistence diagram and related optimum threshold (dashed line); (c) all the cycles displayed with the respective color used in (b); and (d) selected cycle with persistence larger than the optimum threshold.

### 2.3. Images as cubical complexes

Numerical images can be equipped with a topological structure of a simplicial complex equipped with a signal, the intensity, and we can consider the pixels as 0-dimensional pieces, equivalent to Euclidean points. Each couple of pixels is then connected if and only if they share one coordinate; these connections define the 1-dimensional simplices of the space. Finally, the square between connected pixels is a 2-dimensional simplex of the space. This representation defines, in particular, a numerical image as a cubical complex and enables its analysis in the framework of simplicial homology.

To define the persistence diagram of an image, we have to extend the intensity function 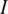 from the set of pixels to the entire complex. This can be achieved by the maximum rule, which consists of assigning the maximum value of its boundary to every simplex of order one or two. For instance, the value of 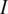 on a connection between pixels corresponds to the maximum intensity value of these pixels. In this way, the intensity function 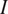 is well defined on the cubic complex, and the associated filtration 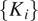 can be correctly defined as specified above.

It is now possible to compute the image’s persistence diagram, containing all the homology class and their persistence. This summarizes the intrinsic topology of the intensity graph and enables looking at shapes within the images in terms of the persistence of related classes. Drawing up the persistence diagram involves considering all the lower-level sets of intensity and constructing a genealogical tree of all classes associated with holes.

To clarify the concepts previously introduced, we spend some words commenting on the example in Figure 1 for the homology of order one ( 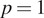 ).

The filtration associated with the lower-level sets defines an increasing family of nested sets for increasing intensity values. The birth of cycles coincides with a local minimum of intensity. In this case, a hole appears in the corresponding lower-level set and defines thus the birth of a new homology class. This class will be kept in the following filtration sets until its death. The intensity value of its death coincides with a local maximum of the intensity. In fact, after this value, the hole merges with the following level sets.

Figure 1 shows the level sets corresponding to local critical points. We observe that three cycles appear at 0. The orange will die at 0.5, while the red will survive until the intensity is 1. The evolution of the green cycle is particularly interesting in this example. In fact, at 0.3, a new cycle appears (blue one), and the original eight shape splits into two circles. However, persistent homology cares about the injection of homology groups and not about the geometric properties of the representative of the homology class. Then, the second hole represents the injection of the green cycle onto the homology group for level 0.3, which does not change its representative. Finally, the levels set at 0.57 and 0.75 show the death of the blue and green cycles, respectively.

The lifespan of each class can be computed using its birth and death values. Then, the persistence diagram is established in Figure 1b, and the related cycles are displayed in Figure 1c.

We also note that two nested shapes (blue and green) are detected. This depends on the saddle point between the two hills that splits the original (green) cycle into two circles (corresponding to the two hills). However, regarding the injection of homology groups, only one new class is born at the saddle point (the blue one), explaining the displayed configuration. We will see in the next section how such a representation can be improved to refine the detection of nested shapes, as shown in Figure 1d.

## 3. Method

This section presents the method used for 3D membrane reconstruction based on the 3D volume (called *z*-stack). As explained below, this is done by computing the persistence diagram of the image and selecting the most persistent classes according to an optimal threshold. The output of this step is a point cloud composed of the segmented contours of each image within the volume. Then, a 3D meshing algorithm is defined to reconstruct a triangulated surface. The method is implemented in Python.

### 3.1. Segmentation

As explained in the previous section, an image can be seen as a cubical complex on which a filtration associated with the intensity function can be defined. Then, we can construct the (relative) persistence diagram and perform segmentation by selecting more persistent homology classes. As membrane profile and cavities appear as cycles (holes) along the filtration, we limit our analysis to the homology of dimension one ( 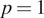 ).

To select automatically the most relevant cycles in a persistence diagram, we introduce the following notion of optimal threshold:

**Definition 3.1 (Optimal threshold).** Let 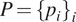 the persistence values associated with points of the persistence diagram. We define the optimal threshold 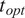 as follows :
(3.1)

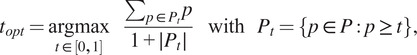

where we suppose that the intensity is normalized to 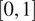 and 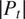 denotes the cardinality of 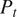 .

The segmentation is based on persistence thresholding and performed via the following steps:

**Membrane profile.** Let 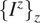 be the set of images contained in the volume. Each image 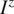 is segmented via a persistence thresholding.

As detailed in the previous section, we can associate a cubical complex 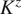 to the image and extend the intensity function 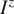 to it. Such an extension, still denoted by 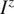 , is defined starting with the values at the 0-simplices (pixels) and by assigning to each 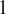 -simplex, and afterward to each 2-simplex, the maximum taken on its boundary.

Then we consider the filtration of the lower-level sets defined as:

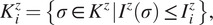

where 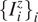 denotes the increasingly ordered set of intensity values. For every 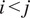 , the injection 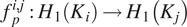 provides information about the similarity of the topological structures for level sets. This enables the establishment of the definition of birth and death values for different classes and the persistence diagram.

The optimal threshold (3.1) is applied to the persistence diagram, which selects the image’s most relevant cycles (see Figure 2).

As we perform the filtration for increasing intensity values, the cycles correspond to white shapes on the dark background. The optimal threshold highlights the most contrasted but can remove lower persistent dark cavities within the membrane profile. For this reason, we consider only the external boundary 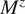 of the shapes detected via persistent thresholding. 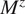 represents the luminal and basal membrane profile, and an independent routine is set up in the next step to detect internal cavities accurately.

**Internal cavities.** This step detects all the cycles inside the membrane profile to reconstruct internal cavities precisely. This task is as challenging as primordial because the lack of ground truth prevents the definition of topological rules for the mesh reconstruction. Obtaining segmentation with well-defined internal holes avoids splitting and branching issues during mesh reconstruction and improves 3D visualization.

In this case, we perform persistence thresholding on the upper-level set filtration

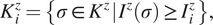

going through the intensity values in descending order. Then, 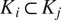 for every 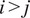 and the homology class of the related homology groups 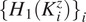 represent now the dark holes within white shapes (like the internal cavities of the cell).

Only the cycles detected within the membrane profile 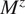 , defined in the previous step, will be considered, which defines the following subfamily of the cycles associated with the filtration:

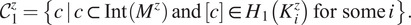



Then, we compute the persistence diagram for all cycles in 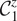 and threshold it using the optimal value defined by (3.1).

**Figure 3.**
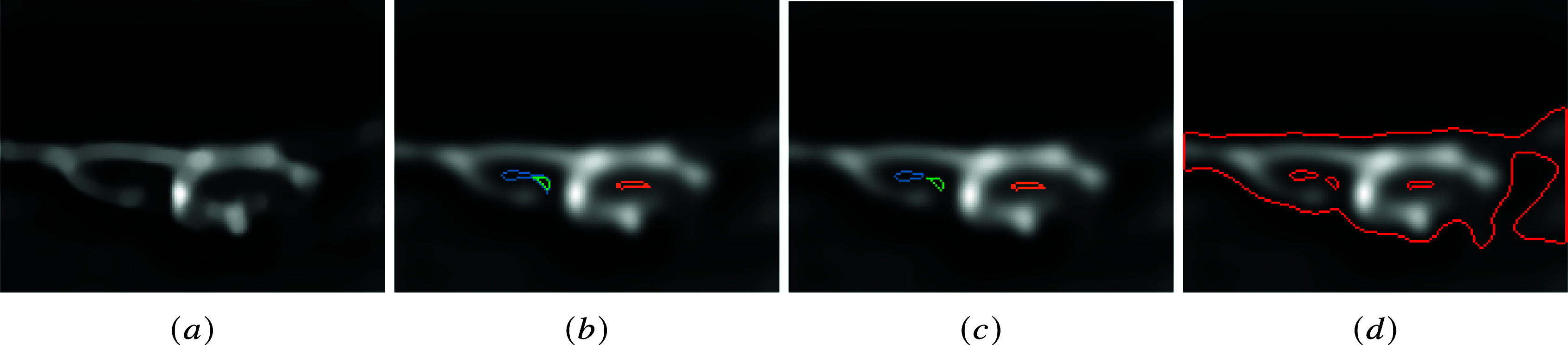
Segmentation of internal cavities: (a) the deconvolved original image; (b) case of nested cycles; (c) separation of nested cycles; and (d) internal holes are drawn with the membrane profile.

Figure 3 shows an example of cavity detection presenting nested cycles. As explained above, separating all cavities and adapting cycle representatives corresponding to saddle points is essential for realistic 3D reconstruction. This is the goal of the last step of the method.

**Nested cycles.** Let 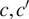 be two planar curves that are representatives of two cycles. We also suppose that their interiors are nested, 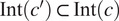 . An example of this configuration is shown in Figure 3 by the blue and green curves.

In this case, we apply a splitting rule based on the filled masks of the cycles, in other words, the interior sets. We perform a morphological dilation of 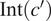 , noted by 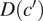 , and we consider the contour 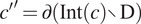 . The two curves 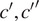 represent now the cycles generated by the saddle point. The directed Hausdorff distance^30^ of 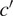 to 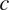 defines the dilation size. This process is applied iteratively to each couple of nested cycles.

Figure 3 shows an example of nested cycles and their new representatives via the previous method. The improved segmentation of the internal cavities is finally displayed with the membrane profile 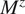 .

### 3.2. Surface reconstruction

The problem of surface reconstruction from planar sections has been extensively studied in image processing and finds several applications in biomedical imaging for 3D visualization. The topological consistency between slices’ segmentations often represents the main challenge, and several methods have been proposed to ensure correct 3D reconstruction.

The primary strategy consists of tiling successive slices’ contours^31^ by a triangulated surface. Several constraints or optimal conditions have been introduced to handle the most general geometries and topologies to guarantee topological consistency between slices. In Refs. 32, 33, the Delaunay triangulation and specific tiling rules generate consistent 3D triangular meshes. In Ref. 34, a partial matching between successive contours is completed to minimize the area of the triangulated surface. In Ref. 35, the reconstruction is achieved by analyzing successive contours overlay. Reference 36 introduced a novel interpolation method to generate evolution between different slices. Moreover, recent methods can handle nonparallel cross-sections and consider topological and geometrical constraints. For instance, Ref. 37 developed an interpolation approach for the user-specified genus, and in Ref. 38, an optimal shape is defined within a family of templates. Finally, in recent years, new methods based on deep learning techniques allow surface reconstruction with finer details.^39^

Maintaining a topological consistency between detected holes within the slices is the main difficulty in our case. This is essential to get a realistic reconstruction of the luminal invagination and internal cavities.

The persistence thresholding and the separation of nested cycles guarantee that all cavities are well separated in slice segmentations. This prevents branching or splitting issues, so a tiling approach is convenient. Moreover, to avoid inconsistent associations, we split each contour’s basal and luminal parts and independently reconstructed the basal and luminal parts of the membrane. We note that, during this splitting, each hole in the segmentation is associated with the luminal part of the membrane.

The surface reconstruction algorithm is based on the following main steps:

**Association problem.** A double association problem is solved between each contour and the successive ones (in the axial direction). First, each point of the contour is associated with its closest point on the same contour. Then, the same point is associated with the closest point living on the successive contour. These associations are constrained by a maximum distance criterion, implying that the optimal point lives in a ball of a given radius around the initial point.

More formally, given two successive contours 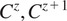 and a maximal distance value 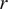 , for every 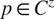 , we solve the following problems

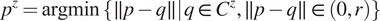




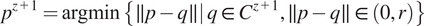

which define the triangle with vertices 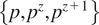 .

The distance criterion is essential to avoid inconsistent connections between the central luminal membrane and cavities sections (holes). This is crucial, for instance, when the first contour contains a hole and the second is a simple curve. In our algorithm, the maximum distance equals 1 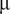 m, corresponding to approximately three pixels for the axial resolution. The solution to this association problem gives a first triangulated surface between a couple of contours, and by iteration on 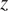 , this defines a triangulation tiling the slices’ segmented contours.

**Smoothing.** We perform mesh smoothing to correct possible curvature artifacts. This can be due to slightly different segmentation in successive slices, introducing curvature in three-dimensional flat shapes. This can be caused by movement during acquisition, unevenness of the signal, or diffusion from neighboring tissue. Mesh smoothing is performed via Taubin’s method^40^ to preserve the final volume of the cell.

**Remeshing.** We finally remesh the triangulated surface to obtain a uniform density of vertices. To this end, we use the clustering-based method developed in Ref. 41.

## 4. Results

This section shows the results of our method applied to confocal data. In Section 4.1, we briefly introduce the biological phenomenon of EHT and the corresponding evolution of cellular morphology. In Section 4.2, the results of the segmentation method are presented, and a comparison with standard methods is performed. Finally, some examples of surface reconstruction are shown.

### 4.1. Biological context

#### 4.1.1. Data acquisition

In vertebrates, hematopoietic stem cells are generated during the embryonic period.^42^ This occurs in vascular tissues of an arterial nature, particularly in the dorsal aorta, where specific endothelial cells (the so-called hemogenic cells) engage in a morphological evolution accompanying their extrusion from the ventral side of the aortic wall. This emergence process, the EHT, is characterized by the very unusual bending of the entire cell toward the subaortic space. After emergence completion, the cells are released and will become hematopoietic stem cells that are at the origin of blood and immune cells of the embryonic and adult bodies.

Imaging techniques enable observing this process *in vivo* in animal models. This process has been described mainly for zebrafish,^27^ ^,^^28^ ^,^^43^ ^,^^44^ for which EHT takes place between 48 hpf (hours postfertilization) and 72 hpf. In particular, confocal-live fluorescence microscopy enables the collection of z-stacks over time to observe this process with a spatiotemporal resolution.^27^

For imaging, embryos were mounted on the top of a glass bottom 60-Dish (35 mm high; Ibidi, Ref. 81156) and embedded on the side position in 1% final low-melting agarose (Promega, Ref. V2111) diluted in Volvic source water and containing tricaine methanesulfonate (MS-222, Sigma-Aldrich, Ref. A5040, 160 μg/ml final concentration). After solidification and before sealing the dish, 1 ml of Volvic source water containing tricaine and N-Phenylthiourea (PTU, Sigma-Aldrich, Ref. P7629, 0.003% final concentration to prevent pigmentation) was added. Embryos were then imaged using an Andor (Oxford Instruments) spinning disk confocal system (CSU-W1 Dual camera with 50 μm Disk pattern and single laser input (445/488/561/642 nm), LD Quad 405/488/561/640, and Tripl 445/561/640 dichroic mirrors), equipped with a Leica DMi8 fluorescence inverted microscope and CMOS cameras (Orca Flash 4.0 V2+ (Hamamatsu)). Imaging was performed using a 40x water immersion objective (HC PL APO 40x/1.10 Water CORR CS2) and a LED light source (CoolLED pE-4000 16 wavelength LED fluorescence system), and the acquisitions were piloted with the support of the MetaMorph software. During acquisitions, embryos were maintained at 28.5 °C using an Okolab cage incubator.

The z-stacks have a resolution of 0.16x0.16x0.3 *μ*m, and acquisition is made every 20 min. We use transgenic fish expressing ras-mCherry recruited mainly to plasma membranes and podocalyxin fused with eGFP recruited at the luminal membrane. Finally, the segmentation method proposed in this work is performed on deconvolved images. For deconvolution, we use the EpiDEMIC plugin available under the Icy software^45^ that performs a 3D blind deconvolution.^46^

#### 4.1.2. State of the art

**Figure 4.**
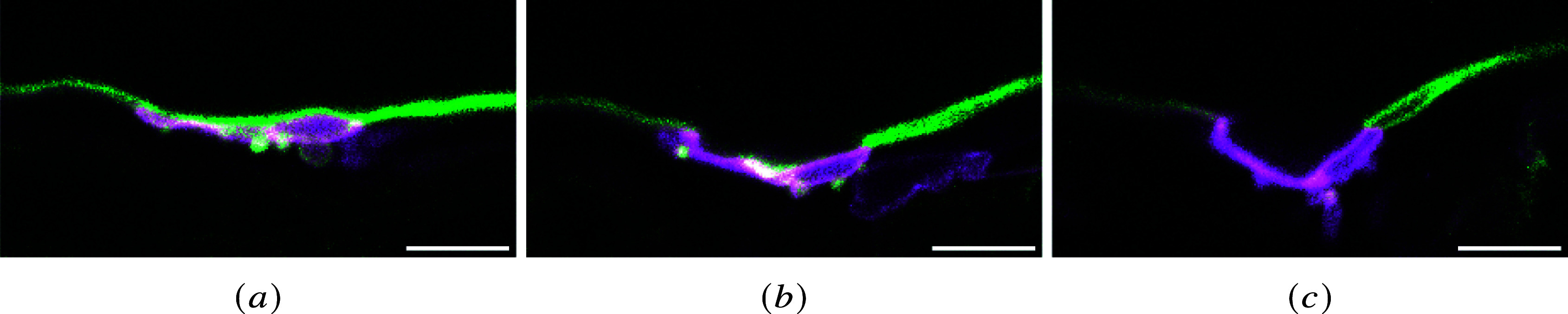
Example of longitudinal sections of the ventral floor of the dorsal aorta (the scale bar corresponds to 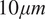 ). From left to right, the figures show three typical sequential stages of the EHT: the flat shape of endothelial cells (a) becomes increasingly curved (b), and the central invagination gradually closes in on itself (c). Magenta: ras-mCherry recruited on plasma membranes; Green: the membrane marker podocalyxin fused with eGPF and enriched in the luminal membrane.

Recent studies^26^ ^–^^28^ reveal that EHT in zebrafish is based on the complex geometrical evolution of cells, with unusual luminal and basal membrane bending toward the subaortic space. The cells initiate the emergence with a relatively flat morphology, bend more and more until the closure of the contact with the aortic lumen, with the ultimate sealing of the aortic floor by adjoining endothelial cells. Figure 4 shows three stages of this evolution. Images also show the presence of blebs on the basal membrane, which confirms the elastic nature of the membrane during the EHT.

Behind this general evolution, the actual geometry of the membrane can be deduced via its longitudinal sections contained in the z-stack. These sections point out the membrane profile, its folds, and the internal voids of the cells. In particular, cavities in the stack images reveal the presence of blebs, cytoplasmic voids, or nonfluorescent structures such as the nucleus (they can be observed next to the basal membrane or at the junctions with other cells). As no topological *a priori* is known about this evolution, accurately detecting these structures in each image is essential to ensure consistent 3D reconstruction.

**Figure 5.**
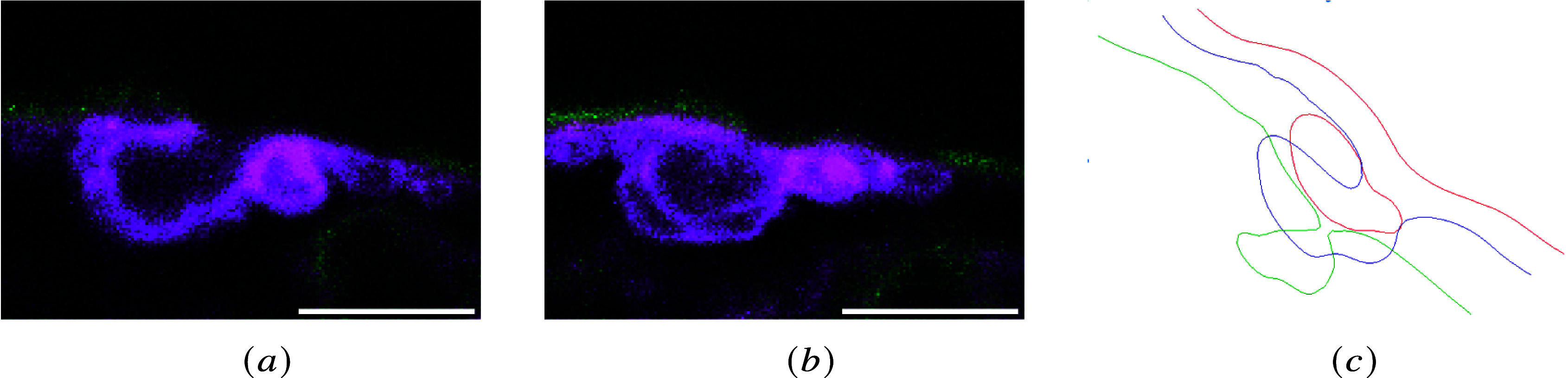
Different images from the same z-stack (the scale bar corresponds to 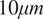 ). (a) membrane profile corresponding to the luminal opening of the invagination of the luminal membrane and a circular cavity corresponding to a bleb; (b) membrane profile showing different cavities below the luminal membrane: the central cavity is a section of the ramified invagination, and the others beneath correspond to cytoplasmic voids; and (c) the cartoon shows a schematic representation of different longitudinal sections of the luminal membrane within the same z-stack.

In Figure 5, some examples of different sections within the same z-stack are shown. The circular opening in the left image corresponds to an invagination of the luminal membrane, whose sections appear as circular cavities (dark inner parts). These cavities are present in the different images of the z-stack, with more or less opening into the aortic lumen. Due to the biomechanical constraints of the tissue and blood flow, the invagination can take surprising shapes with ramifications and inner folds. In the central image, another section of this invagination is visible under the profile of the luminal membrane. The other cavities close to the basal membrane correspond to cytoplasmic voids.

Few pieces of work are specifically dedicated to this type of cell emergence. Campinho et al.^(^^7^ ^)^ studied the influences of flow-dependent mechanical forces on the morphodynamic properties of EHT cells. 3D visualization and cell tracking have been used to collect cell characteristics (number, area, and location) over time and induce a correlation with blood flow intensity.^26^ ^,^^27^ In Ref. 27, an algorithm to unwrap the aorta into 2D cartography was developed to visualize the junctional contacts between emerging cells and their endothelial neighbors. In addition, this article shows that the process progresses over time according to a series of contraction and relaxation phases that are proposed to partly depend on environmental constraints, including forces deployed by endothelial neighbors and the blood flow. Although 2D cartographies allow a global description of the phenomenon via the visualization of intercellular interfaces, a precise 3D reconstruction is essential to visualize and quantify this geometric evolution over time.

### 4.2. Results

**Figure 6.**
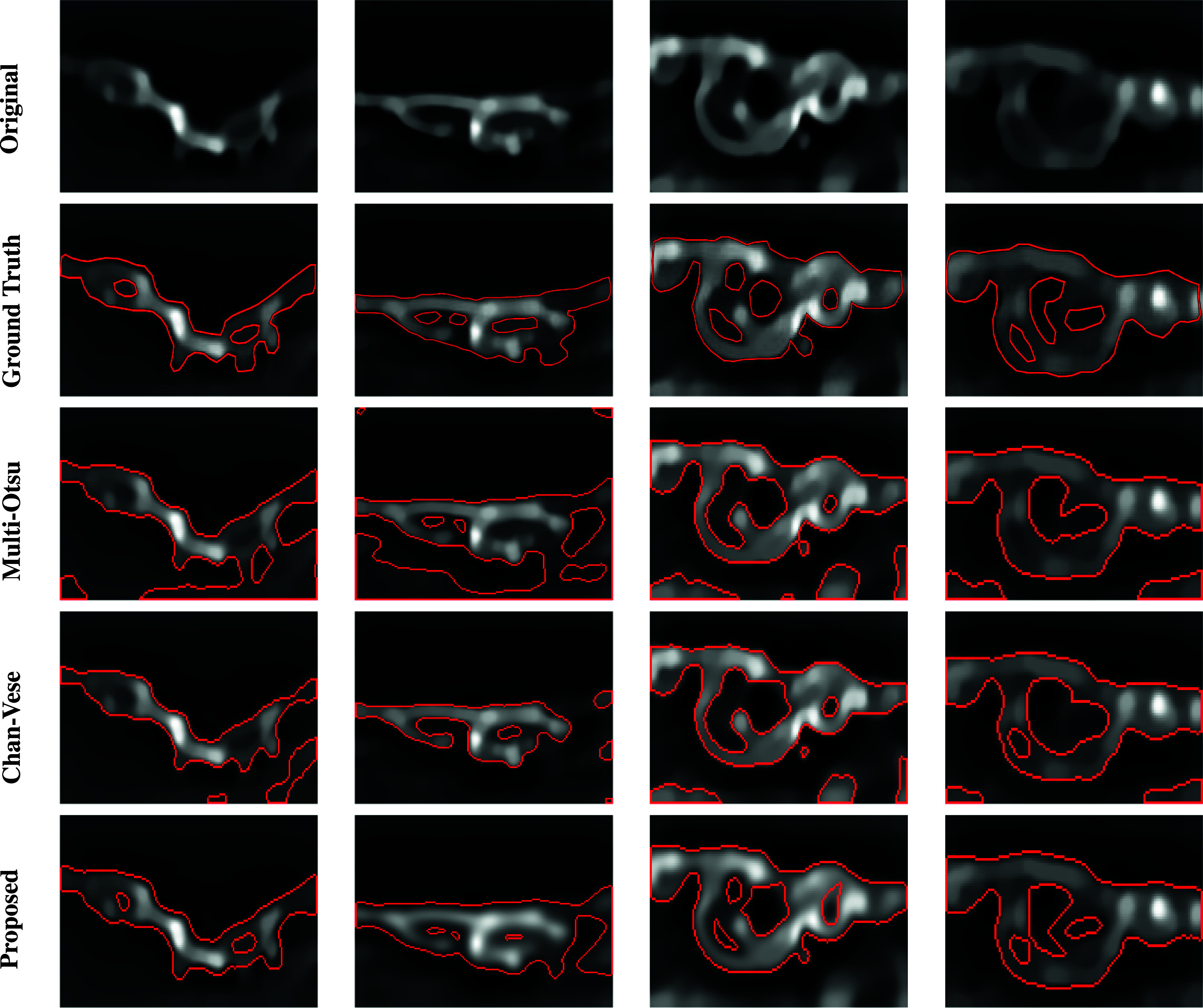
Comparison of segmentation approaches. From top to bottom: deconvolved original image, ground-truth, multi-Otsu thresholding, *^1^* Chan-Vese method, *^3^* and proposed method.

Here, we show some examples of membrane segmentation with the previously defined method. Figure 6 compares with classical methods based on ground-truth segmentation manually defined by biologists.

As specified below, the parameters used for the benchmark methods are optimized, for each method, to minimize the segmentation error over all the images presented in Figure 6.

A first comparison is carried out with the multi-Otsu thresholding method,^1^ which defines several intensity thresholds minimizing the between-class variance criterion. We apply the two-threshold method and choose the lowest one. This method can segment internal cavities if they belong to the level set corresponding to the given threshold, but in reality, cavities can appear at different intensity levels. This is the advantage of persistent homology. The analysis of the topology structure of the intensity graph allows object detection, independently of their specific fluorescence but rather by the relationship of coupled intensity levels with similar topology.

The second comparison is made with the Chan-Vese method^3^ for segmentation. This involves finding a piecewise constant image with distinct constant values inside and outside an optimal curve. The curve and the constant values are defined by minimizing a variational dissimilarity to the original image.^3^ This weighted energy depends on the length of the curve, the area within the curve, and the difference of the images inside and outside the curve. The results in Figure 6 are obtained via the Chan-Vese method implementation in the scikit-image package of Python^47^ with parameters 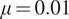 and 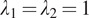 . We point out that the result heavily depends on the choice of the weights for the different terms of the energy. Depending on the shape to detect, these must be adapted, making the method challenging to automatize. In particular, the choice of weights plays a central role in cavities detection. Splitting a curve into several curves might not be optimal for the Chan-Vese energy. This depends on the curves’ lengths and the intensity difference between their inside and outside, so energy weights should be chosen accordingly. Again, persistent homology circumvents this problem with an approach not based on optimal intensity-based criteria.

Beyond the problem of spurious detections, we point out that our method allows the detection of internal cavities and a more geometrically realistic definition of basal and luminal membranes. The topological inspection of the intensity graph is a reliable method to represent the intrinsic structure carried by the signal. This allows one to bypass the definition of intensity-based discriminant criteria for relevant shapes. This represents one of the main advantages of such a method compared to histogram thresholding or intensity-based variational problems. Finally, methods based on persistence diagrams analysis are more robust to intensity variations, need fewer parameters, and are more ergonomic whenever an automatic segmentation of many images is needed. These are the main reasons justifying the use of this approach for our problem, and we believe that it could be helpful in many other imaging processing applications.

For a quantitative comparison with previous methods, we report, in Table 1, the segmentation accuracy calculated on a ground truth that has been manually and collaboratively annotated by expert biologists associated with the work. The entire dataset consists of three newly acquired movies for a total of 20 z-stacks of 40 images each on average (acquisition is made every 20 min). These z-stacks show different cells at different stages of the EHT evolution. For annotation, the biologists independently selected one z-stack per movie, showing different critical stages of the EHT, for a final ground truth of 113 images. They defined the related ground-truth masks by working together and using an Icy^45^ protocol for drawing ROIs. Our method is compared with the two-thresholding Otsu (considering the lowest threshold) and Chan-Vese ( 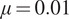 and 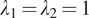 ) methods whose parameters are calibrated on an independent z-stack. We compare the methods in terms of the mean IoU score (Table 1). We recall that the IoU score between the predicted and ground-truth segmentation masks is given by the ratio of the number of pixels contained in the intersection and the union of the masks.

**Table 1.** Methods’ accuracy in terms of IoU score computed on manually annotated ground-truth images

Metrics	Chan-Vese	2-thresholding Otsu	Proposed
IoU (%)	53.6 ± 4.8	63.6 ± 1	72.3 ± 2.6

These results show that the proposed method outperforms the standard approaches to segment the membrane profile and the internal architecture of the cell. As stated above, this is essentially linked to the choice of the parameters; for each of the Otsu and Chan-Vese methods a set of fixed parameters is used for all images, although they heavily depend on the intensity histogram and the *a priori* knowledge of the shapes to be segmented. In terms of computational time, our method is less advantageous than the cited methods. Segmenting an image takes around 1 s by the proposed method for an image of size 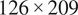 , against 300 ms for the Chan-Vese method and 1 ms for the Otsu method. This is not a major issue for methods used for data exploration and postprocessing routines because the final users do not ask for real time. However, if more rapid results are required, an improved implementation could facilitate its application in real-time analysis.

**Figure 7.**
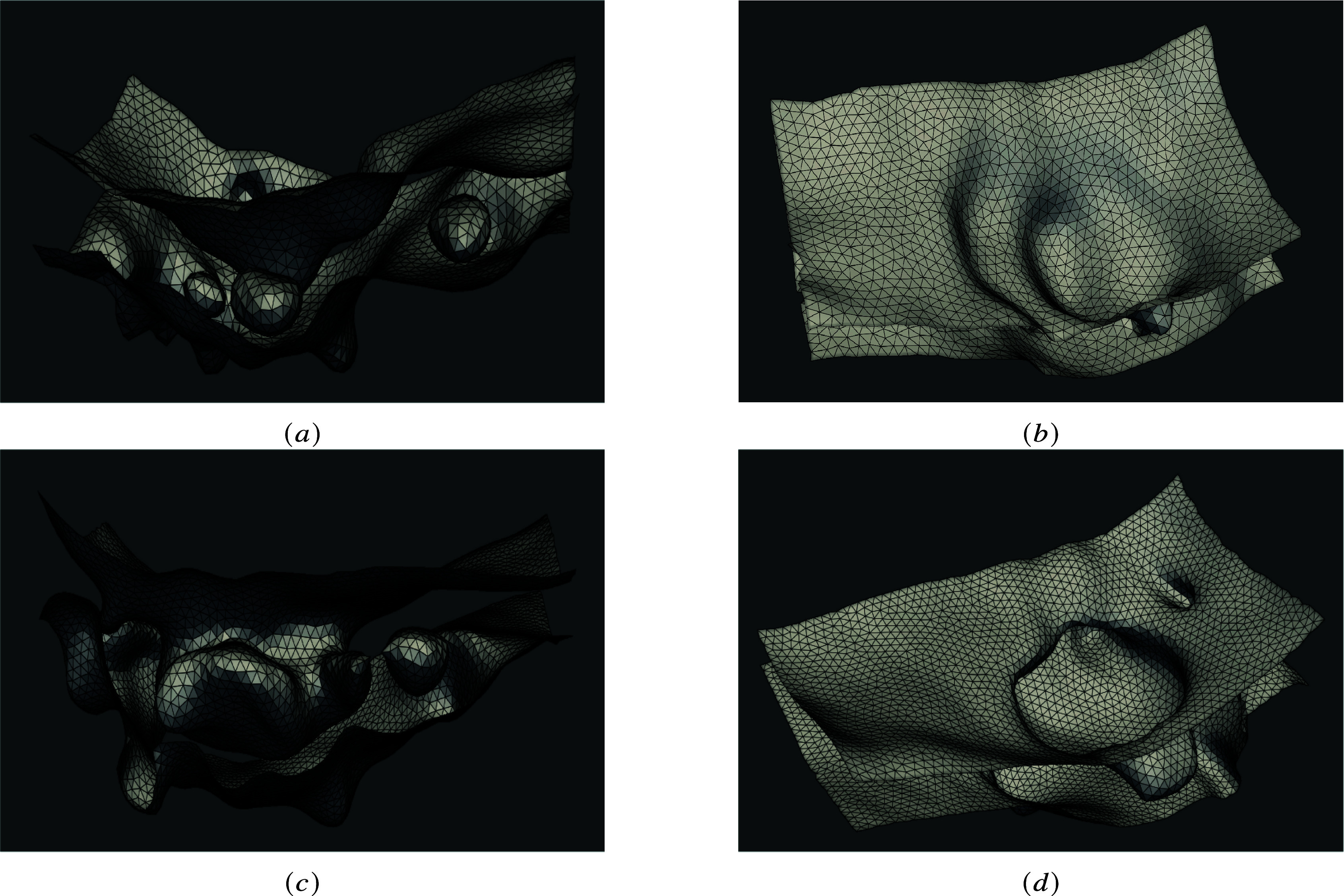
Membrane reconstruction. The meshes corresponding to different stages of the EHT are shown (90 min separate the acquisition timing between the top and the bottom panels). (a, c) a longitudinal view is shown, and we can observe the interior voids and blebs at the basal membrane and (b, d) the luminal part of the cellular membrane is shown, with its quite significant deepening and increased curvature at the rim (see (d)).

Finally, Figure 7 shows an example of membrane reconstruction at different stages of the EHT process. On the left column, a longitudinal view shows blebs on the basal membrane, internal voids, and increasing curvature on the neck of the central invagination. One can appreciate the fine reconstruction of the cell’s internal structures, highlighting nuclei or internal cytoplasmic voids. This enables, in particular, the analysis of cellular organization during EHT.

The right column highlights the luminal membrane invagination with membrane folding and internal ramifications. This points out the elastic behavior of the membrane during EHT and should enable the mechanical analysis of the process over time.

## 5. Conclusions

This article introduces a new framework for 3D image segmentation coupling slices segmentation and mesh reconstruction. In particular, slice segmentation is based on optimal thresholding of the related persistence diagram, enabling the segmentation of the principal topological structures within the image. Moreover, the classical framework of persistent homology is improved to deal with nested cycles and define new, more consistent representative contours.

The method enables object detection with higher precision and topological consistency between slice segmentations. This facilitates, in particular, the mesh reconstruction task without ground truth or related topological *a priori.* Moreover, the proposed method uses a few parameters, the most important (the persistence threshold) being automatically chosen by an optimal criterion. This makes the method more ergonomic to automatize, which is a remarkable advantage when working with a large dataset of images.

The method is applied to an EHT dataset of confocal images, pointing out its benefits compared to classical methods based on clustering or variational techniques. Our approach provides more topologically consistent volume image segmentation, ensuring a realistic 3D reconstruction. For all these reasons, we believe that our method should enable the analysis of the process over time, perform 3D reconstruction of time-lapse sequences, and facilitate biophysical modeling of the related evolution.

Beyond the goal of EHT modeling, this work highlights the power of persistent homology for shape segmentation. This framework, robust to signal unevenness and depending on a few parameters, represents a powerful tool for biological imaging. To our knowledge, this work represents a novel approach to confocal image segmentation, and we hope it serves as an inspiration for other applications.

## Data Availability

The code and data are available at https://github.com/EHTanalysis.
